# Influencing medical student choice of primary care worldwide: international application of the four pillars for primary care physician workforce

**DOI:** 10.1186/s13584-018-0254-8

**Published:** 2018-09-14

**Authors:** Amanda Weidner, Ardis Davis

**Affiliations:** 1 Association of Departments of Family Medicine, Leawood, KS USA; 2 University of Washington, Department of Family Medicine, Box 354696, Seattle, WA 98105 USA

**Keywords:** Family medicine, Primary care, Student specialty choice, Physician workforce, International perspectives

## Abstract

Primary care is a crucial part of a functional health care system, though in many parts of the world there are current or projected gaps in the primary care physician workforce. The academic family medicine organizations in the United States (US) developed the “Four Pillars for Primary Care Physician Workforce,” a model built on decades of research, highlighting four main areas of emphasis for increasing primary care physician output: 1) pipeline; 2) process of medical education; 3) practice transformation; and 4) payment reform. This commentary proposes that this model, although developed in the US context, is applicable in other medical education settings, including Israel, based on the recently reported findings of Weissman and colleagues in this journal.

## Main text

*Author note: We use the term “primary care” to be inclusive; however, in the US and Israel, family medicine physicians make up the majority of primary care physicians, so we use some data referencing “family medicine” in the commentary below.*

It has been well-established that a strong basis of primary care is a critical part of a functional health care system; investing in the design and delivery of primary care as an integral part of the health care system leads to healthier populations [1, 2]. The production of primary care physicians relative to specialists has been declining in the United States (US) [3], and like other parts of the world, including Israel, increased specialization and an aging population are leading to projected workforce gaps for primary care physicians, especially in rural and underserved populations [4–7]. Currently, there are not enough students choosing primary care careers to make up for this projected workforce gap [8].

With this context, the academic family medicine organizations in the US developed the “Four Pillars for Primary Care Physician Workforce,” [9–11] a model that draws on many years of research and highlights the main areas where improvement and innovation may impact the number of primary care physicians in the workforce, and consequently, the health of the public. These Pillars include: 1) pipeline (identifying, recruiting, and retaining students into primary care throughout the continuum of training); 2) process of medical education (excellence in training physicians who practice evidence-based, compassionate, and comprehensive care); practice transformation (exposure to “primary care practices of the future” that deliver evidence-based and patient-centered care); and 4) payment reform (appropriate reimbursement for practice and education). We propose that this model, despite being developed based on the context within and research from the United States, may have components that are generally applicable in other countries, and highlight the applicability to Israeli medical education and health care system, based on the recently reported findings of Naimer [5], Avidan [12], Weissman [13], and colleagues. Components of the Four Pillars model also align well with the expert recommendations suggested by Lahad et al. in their recent commentary reacting to these findings [14].

### Pipeline

Early exposure to medical careers, particularly to primary care, can help increase interest in primary care. Access to role models and ongoing mentoring can help stimulate and maintain interest. Meaningful representation of primary care faculty on admissions committees as well as holistic medical school admissions processes, including consideration of factors associated with higher choice of primary care, such as being oriented to community service and being from a rural background, can help increase admissions of those who are more likely to choose primary care careers [15–17].

These strategies seem universally applicable in places where getting into medical school is a competitive process. Naimer et al. recommend pre-medical mentorship and favoring students interested in primary care careers in the medical school admissions process in Israel. The findings from Avidan et al. builds on this concept of holistic admissions by encouraging marketing primary care differently to various sub-groups of students; for example, focusing differentially on those who have family obligations, have prior exposure to working in healthcare settings, or had previous careers, such as completing military service before medical school in Israel. In the United States, holistic review is increasingly being adopted in admissions and selection which allows for consideration of an applicant’s full story and life path, in addition to quantitative assessments [18].

Weissman et al. recommends marketing in the 4th and 5th years of medical school, referencing data demonstrating that students frequently change their minds about specialty choice before this point. However, given that longitudinal exposure to primary care is the only factor consistently associated with increased proportion of students choosing primary care [19], earlier “marketing” with ongoing support and encouragement – and continued marketing in the later years of schooling – might be better. Many innovative programs have started combining these multiple facets of the *Pipeline* pillar; for example the Targeted Rural Underserved Track at the University of Washington School of Medicine incorporates a special admissions review and selection process, a clinical longitudinal continuity experience tied to a specific rural community, and ongoing support and encouragement over the course of medical school [20].

### Process of medical education

The US differs from Israel and many other countries in its medical education process, where students get a 4-year undergraduate degree before attending 4 years of medical school. At the end of medical school, students participate in a process to match to a residency program; if they do not match in their choice of specialty, students enter a special supplemental match experience to find any open position in their specialty of interest or another “backup” specialty. This contrasts to students in Israel, who start 6-year medical school straight out of high school, and who, as found by Avidan et al., are willing to wait 2–3 years to begin a residency in a specialty of interest if they do not match right away as opposed to immediately starting residency in a specialty that interests them less.

These differences aside, at the medical school and residency levels, learners need excellent and inspiring role models, transformative teaching and experiences, as well as time to explore a diversity of clinical sites and a broad range of clinical care.

The “hidden curriculum” that promotes a hierarchy between specialists and generalists, with the generalist perceived as inferior, has been identified as a factor discouraging students from choosing primary care and family medicine [21]. Israel faces a similar challenge; Naimer et al. reports that students perceive that family medicine suffers from low prestige in the eyes of colleagues as well as the public. Naimer et al. also reports that there is a perception that family medicine is boring with few procedures and suggests exposing students to practices where procedures are common to mitigate this; this suggestion certainly applies elsewhere, including in the US, particularly as scope of practice of family physicians continues to decline [22, 23]. This changing scope of practice is also a potential issue influencing students, as also cited by Lahad et al. in their recent commentary [14], and more study of how this is impacting student choice is warranted.

### Practice transformation

In Israel, as in the US and elsewhere, team care is the “new” model of primary care and becoming increasingly common [4]. Though these teams may not always be comprised of the same types of providers among countries, the concept of teaching medical students to function in interdisciplinary teams by providing education and experiences that involve a variety of learners is paramount to the learning experience and the functionality of these future systems.

Like the US, Israel faces notable discrepancies in the health of different facets of its population [4]. Weissman et al. report that those who are more interested in primary care were more inclined to a specialty dealing with social problems and more interested in residency in the places that most need doctors (i.e. the “periphery”). The cornerstones of primary care, particularly family medicine, are comprehensive, continuity care and practice transformation occurs within this context; the future of primary care practice in the US and Israel, as elsewhere, lies partly in managing the health of populations, including tackling inequalities and addressing the social determinants of health and access to care. Getting students excited about a future career in primary care by embracing and fostering their interest in its social aspects and exposing them to practices that embrace comprehensive continuity care within a team-based model is universal. This is also pointed out by the leaders in the Lahad et al. commentary, and articulated well by Dan Merenstein, MD, who wrote: “lack of procedures, boredom and little action shouldn’t be [the reasons students don’t choose family medicine]” [14].

### Payment reform

Reforming the payment structure of primary care in the US includes not only transitioning from volume-based to value-based care and addressing the gap in reimbursement for primary and specialty care, but also reforming payment for graduate medical education (residency) and addressing student debt, which has a differential impact by specialty. Because of differences in health care system structure and payment, where all OECD countries except the US have the government schemes and compulsory health insurance as the main health care financing arrangements [24], aspects of the “Payment Reform” pillar may be less applicable in other contexts. However, given that international data shows a clear correlation between primary care career choice and the ratio of the mean income of primary care physicians and the mean income of all other physicians [25], reforming payment for primary care has the potential to make the biggest difference in the US [10].

In 2011, Israel implemented sizable salary increases for family medicine physicians as part of a physicians’ union contract and salaries are comparable to other physicians. Additionally, primary care physicians have few on-call obligations and have set hours. Given this, it is unsurprising that Weissman et al. found that those inclined toward primary care were more interested in lifestyle, spending time with their families, working limited hours, and working during the day. Interestingly, however, despite the actual comparability of salaries, Naimer et al. found that there remains a perception that family medicine does not provide a high salary among those not interested in family medicine, though those who were interested in family medicine perceived it to have a reasonable lifestyle to income ratio.

Continued advocacy for payment reforms in the US to support a primary care workforce – and continued advocacy for sharing actual data on family medicine and primary care physician salary and lifestyle in other places like Israel, are critical goals fundamental to increasing the primary care workforce where it is needed.

## Conclusions

Although country-specific approaches to marketing family medicine and primary care in general, as suggested by Naimer et al. are well-supported by the data, the Four Pillars for Primary Care Physician Workforce Reform may be a useful working model for countries seeking to build a strong primary care workforce. For more ideas on what can be done locally, we refer readers to “Putting the Four Pillars for Primary Care Physician Workforce Into Practice Locally” [11].

## Abbreviation

US: United States
